# Biomarkers, the molecular gaze and the transformation of cancer survivorship

**DOI:** 10.1057/biosoc.2013.6

**Published:** 2013-04-22

**Authors:** Kirsten Bell

**Affiliations:** a Department of Anthropology, University of British Columbia, 6303 NW Marine Dr, Vancouver, BC V6 T 1Z3, Canada. E-mail: kibell@mail.ubc.ca

**Keywords:** molecular gaze, molecular biomarkers, cancer, tertiary prevention, risk, subjectivity

## Abstract

Over the past two decades, molecular technologies have transformed the landscape of cancer diagnosis, treatment and disease surveillance. However, although the effects of these technologies in the areas of primary and secondary cancer prevention have been the focus of growing study, their role in tertiary prevention remains largely unexamined. Treating this topic as a problematic to be conceptually explored rather than empirically demonstrated, this article focuses on the molecularisation of tertiary prevention, especially the growing use of molecular biomarkers to monitor disease recurrence. Taking a semiotic approach, I speculate on the potential meanings of molecular biomarkers for people living with and beyond cancer and suggest the meanings of these technologies may differ in important ways for those on both sides of the risk divide: that is, those ‘at risk' for cancer and those living with realised risk. Although molecular biomarkers may intensify a sense of ‘measured vulnerability', by indexing cancer's presence they may also prove reassuring. Moreover, as an invisible but ostensibly ‘transparent' sign, in some contexts they appear to enable cancer survivors to challenge biomedical decision making. In the light of recent oncological debates about the value of these biomarkers in tertiary prevention, I conclude by suggesting that signs can never be reduced to their ‘objective' biomedical denotation in spite of professional attempts to expunge meaning and value from care.

*Between sign and symptom there is a decisive difference that assumes value only against the background of an essential identity; the sign is the symptom itself, but in its original truth.* (Foucault, 1989, p. 115)

## Introduction

Over the past six decades, the rise of cancer screening programmes has transformed the landscape of cancer diagnosis, treatment and disease surveillance. Central to such programmes is the assumption that diseases have latent, early and late manifestations, and that intervention towards the beginning of this natural history can change – or even prevent – an otherwise assured outcome (Armstrong, 2012). Although preventing cancer from emerging in the first place has long been perceived as the ideal, the lack of options for primary prevention led to a focus on early detection in the post-World War II era, which became conceptualised as a form of ‘secondary prevention'. Thus, from the second half of the twentieth century, stopping the disease early by catching it in its latent stage became the goal of cancer screening programmes, although early detection was often conflated with primary prevention in terms of how screening technologies such as mammograms, pap smears and so on were ‘sold' to the population at large (Fosket, 2010, p. 335).

Since the late 1980s, these cancer screening technologies have been joined by a growing array of biomarkers that diagnose cancer – and cancer risk – on a molecular level. The identification of genetic biomarkers of cancer susceptibility has opened up new possibilities for primary prevention, leading to the emergence of tests that screen for genetic carriers of the mutations. It has also stimulated an interest in chemoprevention for those deemed at increased genetic risk for developing cancer; for example, women carrying mutations in the BRCA1/2 genes are now being offered risk-reduction drugs such as tamoxifen (see Eeles and Powles, 2000; Fosket, 2010). The emergence of molecular technologies has also led to a variety of developments in the area of secondary cancer prevention, as proteinic biomarkers of effect (biomarkers associated with possible or established disease) are being widely used to screen for cancer, including prostate-specific antigen (PSA) screening for prostate cancer, cancer antigen 125 (CA125) screening for ovarian cancer and human papillomavirus (HPV) screening for cervical cancer.^1^

Although much of the impetus for the development of molecular screening technologies has related to their possibilities for primary and secondary prevention, with fund-raising campaigns inviting us to imagine a ‘world without cancer', these molecular biomarkers have also increasingly made their way into tertiary prevention as well. Tertiary prevention refers to those aspects of clinical activity involved with reducing the negative impacts of established disease by restoring function and reducing disease-related complications such as cancer recurrence and second primary cancers (Sagar and Lawenda, 2009). Today, proteinic biomarkers are being widely used to monitor patients treated for a number of types of cancer, with regular tumour marker blood tests now an indelible feature of the experience of cancer ‘survivorship' (see Ludwig and Weinstein, 2005). However, as Aronowitz (2009) has pointed out, although social scientists have a growing interest in the expansion of preventive medicine into otherwise healthy populations, far less attention has been paid to parallel developments among the already sick or diagnosed.

There has been a profound, if largely unnoticed, shift in who is understood to be suffering from chronic disease and the disease experience itself. In many instances, chronic disease has become a kind of risk state in which diagnosis, treatment, and ‘disease management' are directed at reducing the chances of anticipated, feared developments. (Aronowitz, 2009, p. 419)

How has the incorporation of molecular technologies in the area of tertiary prevention transformed patients' experience of cancer? If cancer ‘survivorship' has become a kind of risk state in which diagnosis, treatment and disease management are directed at reducing the chances of anticipated, feared developments, can we assume, qua Aronowitz (2009), that the experience of being ‘at risk' for cancer and ‘at risk' for cancer recurrence or disease progression have converged? In my view, this is a phenomenon that needs to be explored rather than assumed.

My goal in this article is to consider the ways in which molecular screening technologies may be impacting the experiences of people with a history of cancer, including both those who have been declared ‘disease-free' and those living with active disease. I am particularly interested in the similarities and differences between those on each side of the risk divide: that is, both those ‘at risk' of cancer and those ‘at risk' of disease recurrence or progression. Through a focus on the semiotic transformations molecular technologies potentially engender, I aim to illustrate some of the ways these technologies differ from those that precede them and how their effects may vary in the contexts of secondary and tertiary prevention. I argue that attempts to limit their semiotic meanings or to pathologise those who ‘misinterpret' these technologies fundamentally misunderstand the nature of signs. I also highlight their socio-political context and the ways in which their ‘appropriate' usage has become tied up with the responsibilities of the ‘good' consumer/citizen.

I should make it clear at the outset that the ideas presented in this article are exploratory and heuristic. Although informed by ongoing research with people who have lived through and beyond cancer, in the present article I treat this topic as a problematic to be conceptually explored rather than empirically demonstrated. My arguments are thus based primarily on a reading of the published literature. However, by offering a semiotically informed analysis, I hope to provide a perspective on these technologies that may benefit social scientists conducting research into this important area.

## Conceptualising Molecular Screening Technologies

The growing incorporation of molecular biomarkers into cancer prevention since the late 1980s has been conceptualised as part of a larger transition that Clarke *et al* (2003, 2010) have labelled biomedicalisation: the ‘increasingly complex, multisited, multidirectional processes of medicalisation that today are being both extended and reconstituted through the emergent social forms and practices of a highly and increasingly technoscientific biomedicine' (2010, p. 47). Through processes of technoscientisation, biomedicine increasingly visualises life at the molecular level in terms of genes, molecules and proteins. This ‘molecular gaze' is enmeshed in a molecular style of thought about ‘life itself', which has seen the body fragmented and reconfigured in new ways (Rose, 2007).

The new ‘molecular biopolitics' (Rose, 2007) engendered by the molecularisation of cancer screening technologies has been the focus of some interest among social scientists, although the majority of research to date has focused on genetic testing, especially in relation to the BRCA1/2 genes (for example, Polzer, 2000; Press *et al*, 2000; Gibbon, 2007; Gibbon *et al*, 2010). Such studies have shown that these predictive tests often serve to fundamentally alter people's experience of cancer risk. As Shostak (2010) notes, individuals with no symptoms of illness are seen as ‘marked' at the molecular level as ‘at high risk' for adverse health outcomes. These tests, in turn, often facilitate new forms of biosociality and somatic individuality as those so labelled start to organise around the commonality of their shared genetic status and the new ‘truths' it inscribes (Rabinow, 1996; Novas and Rose, 2000). For example, people deemed to be at genetic risk for cancer are increasingly conceptualised as ‘previvors':^2^ ‘sur*vivors* of a *pre*disposition to cancer' – a community seen to have its own unique needs and concerns (see FORCE, 2011). To a lesser extent, scholars have also highlighted the forms of embodied risk stemming from elevated PSA levels (Evans *et al*, 2007; Gillespie, 2012) and testing positive for HPV (Kavanagh and Broom, 1997; Aronowitz, 2010) – factors associated with heightened prostate and cervical cancer risk, respectively, but that do not in themselves constitute evidence of the disease. This empirical work speaks to the growing diseasification of risk that molecular screening technologies facilitate, with risk ‘treated' in much the same way as disease itself, through medical means such as medication, behaviour modification and surgical intervention (Armstrong, 1995; Sachs, 1995; Clarke *et al*, 2003, 2010; Aronowitz, 2009; Sulik, 2011; Gillespie, 2012).

Several scholars concerned with processes of biomedicalisation have focused their attention on the semiotic dimensions of visual artefacts associated with ‘things medical' (Clarke, 2010, p. 104), and the social, cultural, political and other contexts in which these artefacts are embedded (for example, Mamo and Fosket, 2009; Clarke, 2010; Joyce, 2010). Situated within this larger framework, in the present article I focus on the semiotic effects of these new molecular ways of visualising the internal workings of the body. As molecular biomarkers are first and foremost a biomedical *sign*, semiotic perspectives have a great deal to offer those interested in the meanings and effects of these technologies.

From a semiotic perspective, all disease monitoring technologies are signs embedded in two types of sign relations: syntagmatic relations and paradigmatic (or ‘associative') relations. Syntagmatic relations are fundamentally sequential: ‘in its place in a syntagma, any unit acquires its value simply in opposition to what precedes, or to what follows, or to both' (de Saussure, 1983, p. 171). A syntagm is thus an orderly combination of interacting signifiers that form a meaningful whole (Chandler, 2007, p. 81). Imaging and molecular technologies form a part of a complex syntagmatic chain, and their value is generated relationally, in the context of the histological, cytological and other data oncologists use to determine diagnostic and prognostic information. However, these technologies are also embedded in paradigmatic relations. Unlike syntagmatic relations, paradigmatic relations are endless, as they acquire their meaning from what is *absent* rather than what is present. They are groups formed by mental association and ‘hold between terms representing a mnemonic group' (de Saussure, 1983, p. 172). It is here that we start to see some important differences between the two technologies.

The distinctions between traditional imaging and newer molecular technologies relate primarily to their differences as sign *types.* According to Peirce's (1931–1958, 2. p. 306) influential triadic classification of sign modalities, there are three types of signs: icons, indexes and symbols^3^. Iconic signs are those in which the relationship between the form the sign takes and its object is based primarily on resemblance (for example, a portrait), indexical signs are those in which this relationship is based on a physical or causal connection (for example, smoke signifies fire) and symbolic signs are those where the relationship is arbitrary – established by convention (for example, language). At first glance, imaging technologies would appear to be indexical signs, much along the lines of photographs. In Peirce's (1998, 2. p. 34) words:

Photographs, especially instantaneous photographs, are very instructive, because we know that they are in certain respects exactly like the objects they represent [i.e., icons]. But this resemblance is due to the photographs having been produced under such circumstances that they were physically forced to correspond point by point to nature. In that aspect, then, they belong to the second class of signs [indexes], those by physical connection.

However, as Dumit (1999) has shown, although imaging technologies such as X-rays and CT (computerised tomography) scans are infused with the persuasive power of photographs, they are more than photographic representations because they purport to represent what no human can see. Thus, as signifiers, their value comes primarily from their perceived likeness to the internal landscape of the body. For this reason, scholars working in the Peircean tradition generally classify imaging technologies as iconic (for example, Nessa, 1996). A suspicious light clump on a mammogram is inspected for its *resemblance* to a malignant tumour; a blob on a CT scan is studied for its *likeness* to an abnormal growth. Molecular technologies, on the other hand, are more clearly indexical in nature: cancerous cells *cause* an increase in particular serum biomarkers; genetic mutations may *cause* cancer to emerge. These biomarkers themselves do not resemble cancer; they index its presence, or, in the case of biomarkers of susceptibility, its potential presence.

Molecular technologies also *quantify* magnitude of disease in a way rather different from traditional imaging technologies.^4^ The main function of mammograms, CT scans and so on is to determine whether cancer is present in the body. In other words, their utility hinges on their ability to ascertain the disease's presence or absence. Thus, although imaging technologies are used in clinical staging, determining the extent of disease before direct examination of the tumour and its spread, this quantification process is not where their primary value resides (and clinical staging is generally perceived as inferior to pathologic staging for this reason). Molecular biomarkers, on the other hand, intrinsically involve a process of quantification. As Shostak (2010, p. 255) notes, molecular practices ‘are used to define a continuum from health to illness with different quantities of markers of “disease accumulation” marking an individual's position and movement along the continuum'. Under a molecular gaze, disease accumulation is not an either/or scenario – one cannot, for example, measure 0 on a PSA test. Indeed, if molecular biomarkers enable scientists to penetrate the silence of the organs (Rose, 2007), it is *numbers* that ultimately allow them to visualise the invisible (Sachs, 1995; Adelswärd and Sachs, 1996). Thus, from a semiotic perspective, the molecular gaze may very well translate to a numeric gaze – a point to which I now turn.

## ‘Measured Vulnerability': Commonalities in Experience Across the Risk Divide

Sulik (2009, 2011), following Clarke *et al* (2003, 2010), has argued that the illness identity has increasingly become a technoscientific one, with many patients beginning to think of themselves in technoscientific terms. Thus, biomedical labels and classifications, rather than their individual suffering, are often determining factors in patients' knowledge synthesis and decision making – a phenomenon that seems equally true of patients' experiences of risk in the realms of primary, secondary *and* tertiary prevention.

On the basis of US research with women and men with elevated cholesterol levels and men with elevated PSA levels, Gillespie (2012) develops the concept of ‘measured vulnerability' to describe the experiences of those people who undergo a shift in their health status as a result of numeric scores on health risk assessments and screenings. He argues that the lack of symptoms associated with elevated cholesterol or PSA levels intensified the pervasiveness of participants' sense of vulnerability, as they were unable to determine their risk without undergoing additional tests. However, these tests only served to intensify uncertainty, creating a vortex of spiralling risk. In consequence, those with elevated cholesterol levels often described themselves as a ‘ticking time bomb' (p. 199), and men with elevated PSA levels expressed a sense of inevitability regarding the eventual onset of prostate cancer. Sachs (1995, 1996; see also Adelswärd and Sachs, 1996) similarly describes the ways in which Swedish men with elevated cholesterol levels came to rely on these tests, which they saw as ‘true' indicators of their health status, regardless of how they actually felt. Abstract test figures thus became a strong focus in their lives and transformed their perception of their bodies, leading to a pervasive sense of felt risk.

In many respects, cancer survivors – particularly those for whom biomarkers feature prominently as a means of monitoring disease recurrence and progression – appear to differ little from people identified as ‘at risk' for disease through biomarker screening. These technologies, and the numbers they generate, are often woven through survivors' accounts of life post-diagnosis in central ways. For example, my earlier ethnographic research with prostate cancer survivors attending a cancer support group suggested that for some men PSA monitoring facilitated a view of prostate cancer cells as lying dormant within them, ready to be reactivated at some future date (Bell and Kazanjian, 2011). Thus, their emotional state became directly tied to their PSA levels, which were seen as a material index of cancer.

This phenomenon has also been frequently noted in anecdotal reports and empirical studies of ovarian cancer survivors, with the CA125 biomarker coming to play a prominent role in many women's experiences of cancer survivorship (for example, Hamilton, 1999; Guppy and Rustin, 2002; Howell *et al*, 2003; Palmer *et al*, 2006; Parker *et al*, 2006; Chitale, 2009). According to Hamilton (1999, p. 339):

Many women begin to identify their CA125 levels as the evidence of disease status …. Unfortunately, even normal insignificant fluctuations in CA125 levels take on enormous meaning. As a result, emotional well-being may come to depend on lower CA125 numbers, even if numbers remain in the normal range. Patients may find themselves on an ‘emotional roller coaster' with ups and downs determined by the direction of serum blood levels.

Cancer listservs attest to a similar phenomenon, with some patients assuming that rising tumour markers in the absence of other signs or symptoms of disease portent a recurrence. For example, in 2010 ‘koe123' wrote on an online testicular cancer forum:

im 26 this yr, early last year i was diagnosed with stage II seminoma which i then went thru 4xBEP [a chemotherapy regimen] and completed it. Since then, everything has been going well as i have always been clear from my check ups and scans until last week. *My blood test result showed a high level of LDH* [a molecular biomarker], *1003 when the normal range is between 100–300.*[…] *i dont know what else can be affecting this elevation other then a relapse*[…] im really freaking out, im praying i dont have to go back into treatment. (TC-Cancer.com, 2011, emphasis added)

After 3 weeks, koe123 reposted that he had completed another blood test, noting that his blood serum levels had dropped but were still outside the normal range. Indicating that this appeared to be a positive development, he nevertheless asked ‘is it true that it is unlikely a relapse?' Striking here is koe123's reluctance to accept that the elevation in his serum levels is meaningless. Signs, as koe123 well knows, must signify *something*.

## The Semiotic Potency of Numbers

What these various accounts allude to is the meaning numbers often come to hold in the lives of those monitored via molecular biomarkers, regardless of whether they are deemed to be at risk for disease *or* disease recurrence or progression. In this context, numbers are imbued with a seductive allure that is difficult to resist.^5^ Numbers, it seems, ‘control the wills of those who make use of them' (Crump, 1990, p. 13). That numerical information becomes an important reference point for cancer patients and patients-in-waiting has been noted previously, albeit primarily in the context of risk and survival estimates. For example, Robertson (2001) documents the preoccupation with numbers among Canadian women at risk of breast cancer, with such women intent on assigning themselves a specific risk figure (for example, ‘a 50/50 chance' of being diagnosed with the disease). A similar fixation with prognostic estimates has been also been documented among cancer survivors (Thorne *et al*, 2006).

Although numbers are symbolic phenomena, they are distinct types of symbols. Unlike other aspects of language, ‘Numerals are clearly weird, atypical of language generally, because the things they denote, numbers, are entities unlike the kind of entities dealt with in the rest of language, say persons, places, things, actions, states and qualities' (Hurford in Crump, 1990, p. viii). There is generally understood to be an isomorphic relation between the structure of mathematics and the structure of reality (Crump, 1990, p. 3), evident in the truism that ‘mathematics is the universal language'. Indeed, Peirce (1894) himself clearly placed mathematical reasoning in the realm of iconic signs, based on the likeness of mathematical equations to phenomena in the natural world (that is, their ability to describe such phenomena). However, for Peirce (1905), this iconicity did not extend to statistics, a field he spoke of rather disparagingly:

A probability … is the known *ratio* of frequency of a specific future event to a generic future event which includes it …. What, then, does it mean to say that if a man sees a phenomenon occur on *m* successive days, the probability is *m*+*1*/*m*+*2* that the same phenomenon will appear on the next following day? …. It plainly means nothing at all of any consequence.

Despite Peirce's disdain, statistics have become a pervasive aspect of our daily lives. As Hacking (1990, p. 4) has observed, the media constantly bombards us with probabilities and statistics; the statistics of our pleasures and vices are relentlessly tabulated and our public fears about cancers, muggings, earthquakes, AIDS and so on are endlessly debated in terms of probabilities. For Hacking (1990, p. 5), the world itself has become numerical; we have gained a fundamentally quantitative feel for nature, both how it is and how it ought to be. More importantly, statistics carry the authority of science. Numbers do not seem arbitrary or biased; their rhetorical effectiveness is facilitated by their apparently neutral non-rhetorical nature and the assumption of calculation as an impersonal, mechanical routine impermeable to human desires and biases (Potter *et al*, 1991, p. 358; see also Porter, 1995; Best, 2001).

Thus, despite their lack of material connection with ‘anything of consequence', statistics have a peculiar power to produce a sense of foreboding and insecurity – ‘statistical panic' in Woodward's (1999) framing. As Woodward (1999, p. 186) notes, ‘If we generally regard statistics as a depersonalizing force … we see that when we apply them to ourselves, creating our own emotional dramas out of them, they can have an overwhelming power, orienting us to the world in a particular way'. Although cancer patients and patients-in-waiting may actively manoeuvre, reframe and discount the odds, they are ‘absorbed into the truth of prognosis, a truth that recursively projects a future as it acts as a container for a present' (Jain, 2007, p. 79). The ‘truth' of prognosis appears ineluctable.

However, semiotically speaking, there are important differences between risk/survival probabilities and the numbers produced in the context of disease monitoring. For the numbers generated through monitoring via molecular biomarkers may be *symbolic*, but they are simultaneously a direct numeric representation of a serum seen to *index* cancer's presence. In other words, the relationship is one of contiguity: cancer's presence causes an elevation in certain proteinic biomarkers. This is not the case for risk or prognostic estimates, which are both strikingly specific and bloodlessly vague (Jain, 2007). After all, you will either be diagnosed with cancer or you will not. And for those diagnosed with the disease, ‘you will only die or not die; you will not 70, or 42, or 97 per cent die' (Jain, 2007, p. 81).

Thus, unlike statistical estimates of risk and prognosis, biomarker counts seem to belong to the world of hard facts: they represent a truth extracted from one's blood (c.f. Biehl *et al*, 2001). Of course, this distinction is more artificial than real. To ‘speak', serum biomarkers are counted and converted into numeric scores – a process, as Martin and Lynch (2009) have shown, that is far from innocent. ‘Techniques for counting co-evolved with the entities being counted and the entities were brought into being as ontologically distinct when they were rendered countable' (p. 248). For example, the understanding of chromosomes as discrete entities and the theories generated about them as hereditary units were constitutively tied up with counting techniques that ‘immobilized, separated and enhanced them' (p. 248). However, the work of counting is rendered invisible and naturalised.

## Realised Risk and Measured Certainty

In the light of these distinctive features of biomarkers, the *meaning* they hold may differ for people ‘at risk' of cancer and those for whom the risks of cancer have been realised – that is, people who have been treated for cancer and who are currently disease-free and those living with active disease. Although such biomarkers may facilitate a new orientation to the body, generating a pervasive sense of ‘measured vulnerability', an important difference is that anxiety and vulnerability are integral to the experience of life post-diagnosis, regardless of the form surveillance takes. Back in 1981 (long before these molecular technologies made their first appearance), Koocher and O'Malley coined the term ‘Damocles Syndrome' to describe the manifestation of this anxiety in its more acute and incapacitating form. As the name of the syndrome suggests, the inspiration for this ongoing sense of embodied vulnerability was the legend of Damocles, who was forced to eat at a magnificent banquet with a sword hanging over his head, suspended from the ceiling by a single hair and poised to plunge into his neck at any moment.

It is little wonder, therefore, that Frank's (1991) concept of the ‘remission society' – those people living in contemporary society who are effectively well but could never be considered ‘cured' – emerged largely from his personal experience with cancer. As Comaroff and Maguire (1981) note, the meaning of the term ‘remission' is profoundly ambiguous, both clinically and experientially. Is the retreat of symptoms partial or total? This condition thus raises problems of meaning; although one may be successfully treated for cancer, one is rarely (except in very specific circumstances) seen as ‘cured'. In consequence, the absence of *symptoms* of disease provides little reassurance regarding the absence of disease itself – after all such absence generally characterises the context of diagnosis in the first place. To quote Jain (2007, p. 80):

Cancer is creepy. After it shows up one realizes that it must have been there for a while, growing, dispersing, scattering, sending out feelers and fragments. After the treatments, often one hasn't any idea if it is still there, slinking about in organs or through the lymph system – those parts of the body you can't really even visualize.

Paradigmatically speaking, absence is semiotically loaded – ripe with meaning. As William James pointed out, the absence of an item determines our representations as much as its presence could ever do (cited in Chandler, 2007, p. 88).

The anxiety absence produces seems to permeate the experience of cancer itself – from diagnosis through treatment, as well as after therapy has ended. Thus, my previous research suggests that cancer patients undergoing chemotherapy may occasionally experience *more* distress from the absence of treatment side effects than from the side effects themselves because of the ways that toxicity is seen to index effectiveness, providing an apparently objective measure of how well the treatment is working (Bell, 2009). In a much earlier study of patient experiences of chemotherapy, Nerenz *et al* (1982, p. 1026) observed an even more curious phenomenon – that patients often became particularly distressed *after* palpable disease signs had disappeared, a response they ultimately interpreted as follows:

When the disease is directly palpable, it has definite size, shape, consistency, and location. Changes in disease status can be directly and objectively monitored so that even an increase in size of palpable nodes can be processed objectively. When the disease is not palpable, however, there is no way to control such emotional states as fear or anxiety about spread of the disease by resorting to objective indices of its change.

Clearly, molecular biomarkers provide such an indices for some patients – albeit one entirely removed from the embodied signs that precede it during cancer treatment (for example, hair loss, flaky skin, reduction in palpable nodes). By marking an individual's position and movement along a continuum, by moving beyond the presence/absence binary, they may prove more reassuring than absence itself.^6^

This sense of ‘measured certainty' (to invert Gillespie's phrase) provided through numeric indices is evident in published accounts of cancer survivorship, with an ovarian cancer survivor quoted in Howell *et al*'s (2003, p. 12) study emphasising the sense of reassurance that accompanies low serum biomarker levels:

… My CA-125 was always under 30. For six years it was under 30. I went from going every 3 months to every 6 months to once a year. For six years I had nothing. I never even thought about it, you know. I never once had a dream about cancer. *I thought I was cured*… *because I was asymptomatic and my CA-125 was always under 30, which means there was no tumor activity going on. Because that always goes up when there's tumor activity for sure.* (emphasis added)

In this woman's case, the feeling of security unfortunately turned out to be false, and she was therefore blindsided by a cancer recurrence; however, that her CA125 levels provided her with a sense of ‘measured' reassurance – ‘for 6 years it was under 30' – is clear.

Evidence of such ‘measured' reassurance is also illustrated in my own research, as the account of ‘Arthur', a 57-year-old Canadian prostate cancer survivor, suggests.^7^ Interestingly, Arthur was not clear on the stage of cancer he had been diagnosed with – ‘He [the oncologist] said I was sort of in the middle …. They didn't catch it really early, they didn't catch it late'. Far more meaningful to Arthur were his PSA numbers, which he made promiscuous reference to throughout the interview. For example, when conversation turned to whether Arthur had received PSA testing since his treatment ended, he responded:

Ah, yeah, and it was very, very low, like 0.02 … it was great, great, great news. And so things look really good right now. But, you know, they take these PSA tests regularly, and what they are watching for is sudden spikes, suddenly it goes up, shoots up. Then there's an indication that there's a problem, if it stays, you know, around 0.02 or 0.04, whatever. Once it was 0.08 and it came back to 0.4, whatever. He [the physician] said, ‘If it changes like that', he said, ‘Your body is going to go through a lot of changes still in years to come. You know this is all going to take years to work its way through your body'.

For Arthur and his physician, his PSA number indexed what was happening inside him. Slight fluctuations in his PSA were a sign of the cancer ‘working' its way through his body and a constantly low number signified that the disease remained at bay. Thus, in response to a question about whether he thought of himself as ‘cured', Arthur stated: ‘So yeah, I think I have the odds in my favour, you know. So, *it's not just wishful thinking*, it's a case of, you know, the way it went, and *the PSA count is so low now, it's looking good. It's looking good, yeah*'. As an ‘objective' sign, his low PSA score reassured him that the possibility of cure was not just ‘wishful thinking'. Arthur's statements suggest that in a context marked by ongoing uncertainty and fear, the semiotic potency of biomarker numbers as transparent, material indices may be substantially heightened.

## Realised Risk and Challenging Biomedical Assessments

Another related feature of biomarker numbers also worth highlighting is the relative clarity they may provide amidst cancer's semiotic ‘din'. Increased diagnostic testing has meant that patients are increasingly caught in an ever-expanding web of tests, with their putative associations between objective signifiers of clinical variation and the probabilities of different diseases (Aronowitz, 2009, p. 429). The language required to understand biomedical technoscience is esoteric, complex and virtually incomprehensible to patients, granting authority to biomedical knowledge and positioning as experts those who produce and communicate it (Sulik, 2011, p. 469). As ‘CancerBaby', blogging about her experience with ovarian cancer, wrote: ‘The vernacular drones constantly …. Rendered mute, you can only listen to the din. It swirls around you, looping endlessly in patterns and figures you can't quite recognize' (quoted in Jain, 2007, p. 78). The diagnosed must work within this paradigm to understand biomedical information, make medical decisions, manage medical interactions and understand their illness experience (Sulik, 2009, p. 1072).

However, although biomarkers are generated in the context of complicated laboratory tests, in some respects this numeric data seems easier to grasp than the other technoscientific information patients are frequently presented with (X-rays, CT scans, biopsies and so on), which must be interpreted by a specialist. One must learn how to read the blurred and shadow-filled landscape of the X-ray – how to distinguish the topography of the body's organs from artefacts introduced during the process of image production. As Dumit (1999, p. 177) observes, ‘X-rays reveal mysteries to experts who alone can explain their meaning'. But despite our much-bemoaned innumeracy,^8^ most of us have general notions about mathematics, tending to see high numbers relating to the body as more risky than low numbers (Adelswärd and Sachs, 1996, p. 1180). Thus, in a sea of floating signifiers, biomarker numbers may provide a reassuringly concrete buoy for patients to cling to, providing a sense of control and empowerment amidst the onslaught of information and decision making that now characterises the ‘career' of the cancer patient (c.f. Aronowitz, 2009).

This attachment to biomarker levels as a way of exerting some control over treatment is evident in several published accounts of the experiences of cancer survivors. For example, an ovarian cancer survivor in Howell *et al*'s (2003, p. 14) study noted:

When I started having the recurrence, I started to feel as though my oncologist wasn't listening. So, in the same way as that in the beginning, I was saying, you know, here are these symptoms and something's wrong. I felt like she [oncologist] was saying, ‘No, it's nothing' or ‘It could be nothing.' So again, I was not feeling that my sense of my own body was being given the credence that it should be. So she would say things like, ‘Well, you know, we don't want to give you chemotherapy. You wouldn't want to have chemotherapy if you don't need it,' kind of. So eventually, *when my CA-125 went up again*, you know, in the fall of last year, . . . *I started to ask about treatments*. (emphasis added)

The apparent objectivity of this numeric sign, with its comparatively unambiguous meaning, provided a means of counteracting her oncologist's dismissal of her subjective sense of dis-ease. Thus, it was when her CA125 marker started to rise that she began to push for treatment.

The semiotic power of biomarkers is also strongly evident in the case of ‘Patricia', a 51-year-old breast cancer survivor and former nurse Sulik (2009) interviewed as part of her research into the creation of technoscientific illness identities. Patricia recounted the following conversation with her oncologist regarding her concerns about metastases:

I said, ‘It [the cancer] could be widespread. We don't know … I haven't had my bone scan, *so I haven't had my tumor markers down* [documented]'. He said, ‘You have far too much knowledge'. I said ‘But, I can't not have the knowledge. You can't suddenly pretend you don't know what you know' (p. 1068).

To the obvious annoyance of her physician, Patricia was not prepared to brook his dismissal of her concerns before documenting her tumour markers; these, rather than his professional opinion, would determine what course of action was warranted.

The role of molecular biomarkers in empowering patients to challenge physician decision making – especially in circumstances where physicians are seen to be overly passive or nihilistic – is evident in the accounts of some cancer survivors in my ongoing research. For example, ‘Jennifer' was a 45-year-old Canadian ovarian cancer survivor treated 3 years earlier with no signs of recurrence at the time of the interview. During the conversation, she recounted how her oncologist had told her at her last visit, ‘well, just so you know, if it comes back there's nothing we can do'. She described her response to this off-hand comment in the following way:

I was caught off guard with this comment because I said ‘Well, what do you mean? Like, *can't we do the CA125 and that would tell me if* – *?*' And she goes, ‘That's – we would only treat you if you're symptomatic'. I said *‘well, if my [tumour marker] levels are quite elevated and, like, there's a laparoscopy, there's all kinds of things'* ….

Evident in these accounts is women's awareness of the hierarchical relationship between signs and symptoms (see Foucault, 1989). Each invokes the sovereignty of the sign over the symptom to advocate a particular course of action. Thus, although the act of discerning the signs of disease via a medical gaze was a way of ‘subtracting' the individual from the picture (Foucault, 1989, p. 15), the dual status of these molecular biomarkers as simultaneously invisible but ‘transparent' signs enables women to reassert individual claims. Notably, instead of generating a sense of resignation or passivity in the face of biological destiny and biomedical expertise, the forms of subjectification facilitated by molecular markers produce in these women an *active* orientation to the future (c.f. Novas and Rose, 2000).

## ‘Duplicitous'^9^ Signs and the Backlash Against Molecularisation

The effects of molecular biomarkers on patients' experiences of life with and beyond cancer are clearly complex, leading in some instances to a new orientation to the body and to the disease itself. However, although such markers have become a standard component of disease surveillance for people diagnosed with a variety of cancers, questions about their reliability remain in the field of biomedicine – and are the subject of ongoing debate. These debates speak to the unevenness of processes of molecularisation – with moments of retreat as well as embrace. As Hogarth *et al* (2012, p. 247) note, ‘the molecularisation of screening may not be a zero-sum game in which new technologies eventually triumph over older ones'.

The recent CA125 controversy is a case in point. The findings of a large randomised clinical trial (Rustin *et al*, 2009) have created intense debate about the role of CA125 in monitoring ovarian cancer survivors. In this trial, those patients treated once they were symptomatic, as opposed to when molecular signs of recurrence manifested (measured via a doubling of serum marker levels), fared no worse – suggesting no survival advantage from intensive CA125 surveillance. Particularly fascinating is the ways this debate about CA125 surveillance came to centre on the semiotic properties of the biomarker. For example, a senior policy director of the Ovarian Cancer National Alliance (a US patient advocacy organisation), speaking out in support of CA125 monitoring, stated: ‘It lets (people) know what is going on in places (they) can't see or feel in the body', expressing her wariness at relying solely on symptoms (Chitale, 2009, pp. 1234–1235). For this woman (echoing the cancer survivors documented above), the ability of biomarker numbers to visualise the invisible is intimately tied up with their value. However, for the director of a gynaecologic oncology programme in Massachusetts, it is precisely this meaning that is the problem: ‘the strong focus on CA125 levels may subtly lead doctors and patients to consider cancer recurrence a numbers game, when it is far more complex' (Chitale, 2009, p. 1234).

The overall consensus to emerge from these discussions appears to be that the technology itself is beneficial when used judiciously – as a sign whose meaning must be evaluated syntagmatically, in relation to a web of other signs and symptoms of disease recurrence. The underlying ‘problem', then, is perceived to be one of patient misunderstanding; patients, in this framing, reify the numbers generated through biomarkers and are thereby guilty of misplaced concreteness. Thus, there is intermittent talk in the literature of ‘CA125 preoccupation' (Parker *et al*, 2006) and even ‘CA125 psychosis'^10^ (Guppy and Rustin, 2002, p. 438; Palmer *et al*, 2006, p. 5) among patients. According to the director of the Massachusetts gynaecologic oncology programme quoted above, ‘Some physicians will treat (a patient) solely on an elevated CA125 with chemotherapy *when patients are upset*' (Chitale, 2009, p. 1234, emphasis added). The underlying discourse here is of the emotional and misinformed patient (and the complicit physician who panders to her whims) inappropriately drawing on health care services – a focus that becomes explicit in the emphasis on health care costs that has accompanied debates about these markers (see Chitale, 2009, p. 1235).

The solution to this ‘problem' of patient ‘misunderstanding' of biomarkers? Patient education: the now-universal response to any evidence of a disjuncture between patient and physician perspectives on health, risk and illness (see Parker *et al*, 2006). Thus, the prevailing view is that ovarian cancer survivors should be ‘informed about the usefulness and drawbacks of CA125 measurements' so they can make an ‘informed choice' about being monitored through the biomarker (Karam and Karlan, 2010; see also Fayed, 2009). This is, of course, exactly the rhetoric that surrounds PSA screening – a test similarly marred by controversy surrounding its utility in secondary cancer prevention.

However, a brief look at PSA screening demonstrates the limitations of the patient education model. Despite the controversy surrounding PSA screening, it is an extremely common test; for example, although it is not recommended in the Canadian guidelines, almost 50 per cent of Canadian men over 50 report being screened at some point (Beaulac *et al*, 2006). Indeed, studies have found that men still view the PSA test positively once they have been ‘informed' of its limitations (Hewitson and Austoker, 2005; Watson *et al*, 2006). It therefore seems reasonable to surmise that if the opportunity for CA125 monitoring exists most ovarian cancer survivors would choose to avail themselves of it, regardless of any stated limitations. Indeed, patients priorly monitored through CA125 are often quite distressed when their access to the test disappears, as has happened in some clinical settings in the United Kingdom since the debates about CA125 monitoring first occurred (Amy Ford, personal communication).

This attempt to limit the semiotic meanings of biomarkers via patient education (a version of the ‘sometimes a cigar is just a cigar'^11^ lecture, one presumes) fundamentally misunderstands the nature of signs. Physicians' underlying assumption appears to be that new technologies can be introduced without implications for patient experiences of disease – that signs can be reduced to their ‘objective' biomedical meanings. Beyond the impossibility of limiting semiosis in this way, it speaks to the ongoing inability of biomedicine to deal with *meaning*; that is, its continual turn away from illness in favour of disease (Kleinman, 1988). To quote Kleinman (1988, p. 30), ‘questions of the cultural significance of risk as bafflement come to the fore in spite of professional (and societal) attempts to expunge meaning and value from the equation of care'.

It also elides the ways in which patients have been transformed into ‘medical consumers', expected to take charge of their health through proactive and prevention-conscious behaviour, rationality and choice (Sulik and Eich-Krohm, 2008). In this framework, screening becomes tied up with the responsibilities of the ‘good' neoliberal consumer/citizen (Rose, 1993, 1999; Clarke *et al*, 2003, 2010). Molecular technologies thus operate in a political and ethical field in which individuals are obliged to formulate life strategies, maximise their life chances, take actions (or refrain from them) in order to increase their quality of life and to act prudently in relation to themselves and others (Novas and Rose, 2000, p. 487). The pursuit of health is both a civic and individual duty. Thus, ideal ‘healthy' citizens take in screening procedures such as cervical pap smears and blood cholesterol tests – but only, of course, as appropriate (Petersen and Lupton, 1997). Pathological risk management among the ‘worried well' is to be discouraged (Wagner and Curran, 1984), the ideal citizen being one who manages risk in a cost-effective way. ‘Good' patients are required to act in a similar fashion – to recognise their vulnerability and take steps to manage their risks of disease recurrence via regular monitoring and surveillance, but not to use these technologies ‘inappropriately', in a cost-inflationary fashion.

## Conclusions

If processes of molecularisation have transformed the face of primary and secondary cancer prevention, engendering new forms of somatic individuality in asymptomatic populations, their impact in the realm of tertiary prevention is no less fundamental (albeit far more overlooked). Examined from a semiotic perspective, there are important differences between these molecular technologies and the array of imaging technologies they now supplement. Thoroughly indexical, these technologies serve to *quantify* disease along a continuum, potentially producing an orientation to the self that is as much mathematical (or numeric) as molecular. Numbers, with their apparent transparency and neutrality, become a powerful lens through which experience is filtered. This is particularly true of the biomarkers used in tertiary prevention, which some cancer survivors perceive as a material index of cancer's presence in the body.

While this numeric gaze seems to develop on both sides of the risk divide, with those ‘at risk' of cancer and those ‘at risk' of cancer recurrence or progression each developing a sense of measured vulnerability, I have suggested that there are some potentially important differences in the *meaning* of these technologies for those living with realised risk. Although molecular biomarkers may create a sense of measured vulnerability in cancer patients, by making the invisible visible they are simultaneously a reassuring sign – potentially more reassuring than absence itself, given its semiotic potency. Moreover, their apparent simplicity and transparency may also serve to provide a sense of control and empowerment, enabling cancer survivors to challenge medical decision making and generating an active orientation to the future.

That said, I do not intend to suggest that the effects of these technologies are universal or uniform. Certainly, some cancer survivors I have interviewed refer in vague terms to ‘markers' and ‘blood tests', placing no particular significance on numeric scores. Sulik (2009) is careful to point out that patients who go on to develop technoscientific illness identities may incorporate them in a partial rather than complete way. According to Clarke *et al* (2010, p. 82), these identities may also be invoked strategically, seemingly accepted to achieve particular goals (for example, advocating for a particular course of action), but in other situations also refused. There is potentially also a strong class dimension to the ways these technologies are taken up (or not) among cancer patients; as Crawford (1980) noted in his original formulation of ‘healthism', the middle class are more likely to subscribe to the notion that health and disease are situated at the level of the individual, and to embrace the norms of enterprising and responsible personhood that it assumes.

However, there is danger in ignoring the semiotic dimensions of these technologies – or, worse still, assuming that they can be reduced to their biomedical meaning and pathologising those for whom a number becomes more than just a number. For a cancer survivor, is a cigar ever just a cigar? (Or a headache ever just a headache, or a sore joint ever just a sore joint?) As Staiano-Ross (2007, p. 37) observes:

[T]he body that produces signs or from which signs are produced is a ‘knowing' body, one which can never be separated entirely from the social and political milieu within which it exists … I cannot extract my body from this … I visualize my body in ways that are determined not by my having actually seen inside my body, but in ways I am shown that the body must be. These are fully embodied attitudes and perceptions. The signs of illness are not simply indices that point to an object within the body, but symbolic ‘outputs' of complex processes.

For these reasons, semiotically informed approaches have the potential to provide new insights into patients' understandings and experiences of molecular screening technologies and to redress the limited analytic scope of much current research into the sociology of screening (see Armstrong and Eborall, 2012). While it is widely accepted that the ‘molecular gaze' has transformed and reconfigured understandings of the body, semiotics helps us to explicate the *nature* of the differences between molecular screening technologies and older methods of visualising the internal body. If, as I have speculated in this article, the molecular gaze translates into a numeric gaze, we must more clearly grapple with the meaning of biomarker numbers for patients. As I have tried to show, semiotically speaking, these numbers differ in fundamental ways from the statistics and probabilities that precede and accompany them. Instead, they purport to speak a more direct and personal truth: one taken directly from the patient's own blood. Although the implications of this shift require further study in the realm of cancer, they also require consideration and analysis in the context of the growing array of chronic conditions where molecular technologies are being developed to monitor disease status, from HIV/AIDS (Price *et al*, 2007) and diabetes (Rossing *et al*, 2008), through to neurological disorders (Mayeux, 2004) and cardiovascular diseases (Vasan, 2006).

## Footnotes

1 Although the PSA test is widely available, its use as a screening tool remains controversial, with the originator of the test condemning its role in cancer screening based on available evidence that PSA screening does not lead to fewer deaths from prostate cancer but leads to substantial over-diagnosis and over-treatment of the disease (Ablin, 2010). CA125 is not currently recommended for population-level screening, but the National Institute of Health and Clinical Excellence, the organisation responsible for determining clinical and public health guidelines in the United Kingdom, has recently (and controversially) recommended the use of the test in primary care settings for women who present with symptoms associated with ovarian cancer (see Olaitan, 2011). Women concerned about ovarian cancer can also request the test (see Fayed, 2009). HPV testing is a standard component of cervical cancer screening, along with the pap smear itself.

2 The implicit referent for the term ‘previvor' is ‘survivor', suggesting a paradigmatic connection between these two groups, which is, of course, precisely Aronowitz's (2009) point.

3 Importantly, these modes of relationship are ideal types; in reality, signs partake of all three qualities to varying degrees (Peirce, 1931–1958, 2. p. 306).

4 This holds less true for some of the newer ‘imaging' technologies, such as functional magnetic resonance imaging, which is more accurately classed as a molecular technology. As Roskies (2007) demonstrates in her work on neuroimaging, although these images are understood as photographs of brain activity, what they are sensitive to is not visual information but magnetic signals.

5 Interestingly, this phenomenon is also evident in MacIntyre's (1999) study of the emotional role of T cells counts in the lives of men living with asymptomatic HIV. For men who initially tried to avoid playing the numbers game, once they succumbed to testing, ‘retreat was out of the question' (p. 133).

6 I freely acknowledge that this largely contradicts my prior reading of PSA monitoring (see Bell and Kazanjian, 2011).

7 This ongoing research includes interviews with Canadian cancer ‘survivors' on their experience of living with and beyond cancer and the ways they relate to dominant biomedical and cultural discourses on the disease. This research has not focused specifically on participants' understandings of molecular biomarkers, but it is a topic that has frequently come up in certain interviews, especially among prostate and ovarian cancer survivors.

8 See, for example, Paulos (1998). However, the extent of our innumeracy is debated, with some researchers pointing to evidence of quite sophisticated mathematical calculations routinely made by ‘plain folks' engaged in supermarket shopping (see Crump, 1990, p. 21).

9 See Karam and Karlan (2010) for a discussion of the ‘duplicity' of CA125 measurement.

10 In the light of the fact that similar issues plague PSA monitoring, one cannot help but wonder whether this pathologisation of patients deemed to become overly ‘preoccupied' with their biomarker levels is implicitly gendered.

11 This statement is often attributed to Sigmund Freud, although evidence suggests that he probably did not make it (see Quote Investigator, 2011). Indeed, it seems quite out of character for someone so centrally attuned to symbolism.
